# Editorial board

**DOI:** 10.1080/0886022X.2026.2616071

**Published:** 2026-02-03

**Editor-in-Chief**

**Tibor Fülöp,** MD, PhD (Professor) - Medical University of South Carolina, Charleston, South Carolina, USA

**Deputy Editors**

**Mihály Tapolyai,** MD, PhD (Nephrologist) - St. Margit Hospital, Budapest, Hungary and Ralph H. Johnson Veterans Affairs Medical Center, Charleston, South Carolina, USA

Wisit Cheungpasitporn, MD (Professor) - Mayo Clinic, Rochester, Minnesota, USA

**Social Media Editor**

**Wisit Cheungpasitporn,** MD (Nephrologist) - Mayo Clinic, Rochester, Minnesota, USA

**Senior Associate Editors**

- **Andrea Angioi,** MD (Nephrologist) - ‘Giuseppe Brotzu’ Hospital, Cagliari, Italy
- **Monika Gööz,** MD, PhD (Associate Professor) - Medical University of South Carolina, Charleston, South Carolina, USA
- **Fan He,** MD, PhD (Professor) - Tongji Medical College, Huazhong University of Science and Technology, Wuhan City, Hubei Province, China
- **Wisit Kaewput,** MD (Associate Professor, Nephrologist, Lecturer and Deputy Head of Department) - Phramongkutklao Hospital and Phramongkutklao College of Medicine, Bangkok, Thailand
- **Csaba Kopitko,** MD, PhD (Anesthesiology & Critical Care Specialist) - Hospital of Hungarian Armed Defence Forces, Budapest, Hungary
- **Guisen Li,** MD, PhD (Chief Physician) - Sichuan Academy of Medical Sciences and Sichuan People’s Hospital, Chengdu, Sichuan, China
- **Ákos G. Pethő,** MD, PhD (Assistant Professor) – Semmelweis University, Budapest, Hungary
- **Jianbo Qing**, MD (Researcher) - Sir Run Run Shaw Hospital, Zhejiang University, Hangzhou, China
- **Karim M. Soliman,** MD, PhD (Associate Professor) - Medical University of South Carolina, Charleston, South Carolina, USA
- **Charat Thongprayoon,** MD (Assistant Professor and Senior Associate Consultant) -Mayo Clinic, Rochester, Minnesota, USA
- **Ningning Wang,** MD, PhD (Professor) - The First Affiliated Hospital of Nanjing Medical University, Nanjing, China
- **Guangtao Xu,** MD (Researcher) - Jiaxing University, Jiaxing, Zhejiang, China
- **Zhongheng Zhang**, MD (Nephrologist) -Sir Run Run Shaw Hospital, Zhejiang University School of Medicine, Hangzhou, China

**Associate Editors**

- **Nicola Lepori,** MD (Assistant Professor) - University of Cagliari and ARNAS Brotzu, Cagliari, Italy
- **J. Pedro Teixeira,** MD (Assistant Professor) - University of New Mexico, Albuquerque, New Mexico, USA

**Biostatistics and Statistical Consultant Advisor**

**Zsolt Lengvárszky,** PhD (Professor & Department Chair) - Louisiana State University Shreveport, Shreveport, Louisiana, USA

Section Editors*

***Acute Kidney Injury***

**Jonathan Samuel Chávez Iñiguez**, MD (Chief of Nephrology) - Universidad de Guadalajara, Guadalajara, Mexico

**Hongqi Ren,** MD, PhD (Director) - Nanjing University School of Medicine, Nanjing, China

**Bijin Thajudeen,** MD (Professor & Deputy Chair of Medicine) - Banner University of Arizona, Tucson, Arizona, USA

***Anemia and Hematologic Disorders***

**A. Emre Eşkazan,** MD (Professor) - Istanbul University-Cerrahpaşa, Istanbul, Turkey

***Artifical Intelligence and Machine Learning***

**Hatem Ali,** MBBCh, MSc (Clinical Research Fellow) - University Hospitals of Coventry and Warwickshire, Coventry, UK

***Basic Sciences Investigations***

**Ahmed I. Adelkader**, MD, PhD (Transplant Nephrology Fellow) - Medical University of South Carolina, Charleston, South Carolina, USA and Mansoura University, Mansoura, Egypt

**Alejandro R. Chade,** MD (Professor and NextGen Precision Health Investigator) - University of Missouri-Columbia, Columbia, Missouri, USA

**Natalia Stepanova,** MD, PhD, DSc. Med (Professor) - Institute of Nephrology of the National Academy of Medical Sciences of Ukraine, Kyiv, Ukraine

***Bone-Mineral and Electrolyte Disorders***

**Chen Fu,** MD (Nephrologist) - China Beijing Jishuitan Hospital Capital Medical University, Beijing, China

**Gheun-Ho Kim,** MD, PhD (Professor) - Hanyang University College of Medicine, Seoul, Korea

***Cardio-renal Physiology and Disease Processes***

**Alejandro R. Chade,** MD (Professor and NextGen Precision Health Investigator) - University of Missouri-Columbia, Columbia, Missouri, USA

***Chronic Kidney Disease and Progression***

**Sebastjan Bevc,** MD, PhD (Professor) - University Medical Center Maribor and University of Maribor, Maribor, Slovenia

**Xuxia Gao,** MD, PhD (Nephrologist) - Capital Medical University Affiliated Beijing Anzhen Hospital, Beijing, China

**Fan Yi,** PhD (Professor) - Shandong University, Jinan, Shandong, China

***Critical Care Nephrology and Continuous Kidney Replacement Therapy***

**Jorge Castaneda,** MD (Critical Care Specialist) - Advent Health New Smyrna Beach Hospital, Florida, USA

**Abduzhappar Gaipov,** MD, PhD (Professor) - Nazarbayev University School of Medicine, Nur-Sultan City, Kazakhstan

***Glomerulonephritis and Immunologic Disorders***

**Ana de Lurdes Agostinho Cabrita Vieira,** MD (Nephrologist) - University Hospital Center of Algarve, Faro, Portugal

**Stefan Gabriel,** MD, PhD (Professor) - Carol Davila University of Medicine and Pharmacy, Bucharest, Romania

**Nicolas Hanset,** MD (Nephrologist) - University Hospital Saint-Luc, Brussels, Belgium

***Hemodialysis and Peritoneal Dialysis***

**Gaetano Alfano,** MD (Nephrology Medical Director) - University Hospital of Modena, Modena, Italy

**Jin-Bor Chen,** MD (Nephrologist) - Kaohsiung Chang Gung Memorial Hospital, Chang Gung University College of Medicine, Kaohsiung, Taiwan

**Surapon Nochaiwong,** PharmD (Lecturer) - Chiang Mai University, Chiang Mai, Thailand

***Hypertension and Volume Management***

**Ana de Lurdes Agostinho Cabrita Vieira,** MD (Nephrologist) - University Hospital Center of Algarve, Faro, Portugal

**S. Mehrdad Hamrahian,** MD (Professor) - Thomas Jefferson University, Philadelphia, Pennsylvania, USA

**Gheun-Ho Kim,** MD, PhD (Professor) - Hanyang University College of Medicine, Seoul, Korea

***Imaging Technologies in Nephrology***

**Alejandro R. Chade,** MD (Professor and NextGen Precision Health Investigator) - University of Missouri-Columbia, Columbia, Missouri, USA

***Medical Education and Training***

**Yavuz Ayar,** MD (Associate Professor) - University of Health Sciences, Bursa Faculty of Medicine, Bursa, Turkey

***Nephrolithiasis and Urolithiasis***

**Natalia Stepanova,** MD, PhD, DSc.Med (Professor & Chair) - Institute of Nephrology of the National Academy of Medical Sciences of Ukraine, Kyiv, Ukraine

***Nephrology Nursing and the Patients’ Voice***

**Amy Perry,** MSN, APRN, BC, CNN-NP 9 (Nurse Practitioner) - Ralph H. Johnson Veterans Affairs Medical Center, Charleston, South Carolina, USA

***Pediatric Nephrology***

**Sihem Darouich,** MD, MSc (Professor) - University of Tunis El Manar, Tunis, Tunisia

***Pharmacology and Nephro-Oncology***

**Jorge Castaneda,** MD (Assistant Professor) - Advent Health New Smyrna Beach Hospital, Florida, USA

**Christian A. Koch,** MD, PhD, Medhabil, MACE (Professor and Section Chief) - University of Florida, Jacksonville, Florida, USA

**Jolanta Malyszko,** MD, PhD (Professor and Department Chair) - Medical University of Warsaw, Warsaw, Poland

***Transplantation***

**Orsolya Cseprekál,** MD, PhD (Senior Lecturer) - Semmelweis University, Budapest, Hungary

**Miklos Z. Molnar,** MD, PhD (Professor and Medical Director) - University of Utah, Salt Lake City, Utah, USA

**Ken Sakai,** MD, PhD (Professor & Chairman) - Toho University Faculty Medicine, Tokyo, Japan

*The Section Editors act as Topic Advisors to the editor and associate editor team and do not handle papers directly unless they are also listed as Associate Editors

**Editorial Board Members**

- **Moh’d Mohanad A. Al-Dabet**, PhD (Associate Professor) – American University of Madaba, Amman, Jordan
- **Abdullah K. Al-Hwiesh,** MD (Professor and Consultant) - Imam Abdulrahman Bin Faisal University, Al-Khobar, Saudi Arabia
- **Petar Avramoski,** MD, PhD (Professor) - St. Clement of Ohrid University, Bitola; Clinical Hospital – Bitola, North Macedonia
- **Mickaël Bobot,** MD, PhD (Nephrologist) - Aix Marseille Université, Marseille, France
- **Tiffany N. Caza,** MD, PhD (Nephropathologist and Scientist) - Arkana Laboratories, Little Rock, Arkansas, USA
- **Jonathan Ciofani,** MD, BMedSc, MPH - Sydney Medical School, The University of Sydney, Sydney, Australia
- **Ahmed Daoud,** MD, PHD (Lecturer) - Cairo University, Cairo, Egypt
- **Shao-bin Duan,** MD, PhD (Chief Physician and Professor) - The Second Xiangya Hospital of Central South University, Changsha, China
- **Wayne Fitzgibbon,** PhD (Associate Professor) - Medical University of South Carolina, Charleston, South Carolina, USA
- **Winston Wing-Shing Fung**, MBBChir (Associate Consultant and Honorary Assistant Professor) - Chinese University of Hong Kong, Hong King, China
- **Mohamed Hassanein,** MD, FNKF, FASN, FACP (Consultant Nephrologist) - Kidney and Urology Center, Alexandria, Egypt
- **Quan Hong,** PhD (Nephrologist) - Chinese PLA General Hospital, Beijing, China
- **Yuh-Shan Ho,** PhD (Director and Associate Professor) - CT HO Trend, Tapei, Taiwan
- **Ivica Horvatic,** MD, PhD (Nephrologist) - Dubrava University Hospital, University of Zagreb, School of Medicine, Zagreb, Croatia
- **Bo Hu,** MD (Associate Professor) - Jiaxing Hospital of Traditional Chinese Medicine, Jiaxing University, Zhejiang, China
- **Jun Jiang,** MD (Nephrologist) - The First Affiliated Hospital of USTC, University of Science and Technology of China, Hefei, China
- **Haobo Li,** MD (Researcher) - University of Bordeaux, Bordeaux, France
- **Yafeng Li**, MD, PhD (Professor and Director) - Shanxi Medical University, Taiyuan, China
- **Qiu Li,** MD, PhD (Nephrologist) - Children’s Hospital of Chongqing Medical University, Chongqing, China
- **Tongqiang Liu,** PhD (Nephrologist) - Nanjing Medical University, Changzhou, Jiangsu, China
- **Alisson D. Machado,** PhD (Professor) - University of Sao Paulo, Sao Paulo, Brazil
- **Shan Mou,** MD, PhD (Professor and Director Physician) - Shanghai Jiao Tong University, Shanghai, China
- **Lovelesh Nigam,** MD (Associate Professor) - Institute Of Kidney Disease And Research Center, Dr. H.L. Trivedi Institute of Transplantation Sciences (IKDRC-ITS), Asarwa, Ahmedabad, India
- **Yannick Nlandu Mayamba,** MD (Nephrologist) - University of Kinshasa, Kinshasa, Democratic Republic of the Congo
- **Huiwen Ren,** MD, PhD (Associate Professor) – Dalian Medical University, Dalian, Liaoning, China
- **Vinayak S. Rohan,** MD (Associate Professor of Surgery) Northwestern University, Chicago, IL, USA
- **László Rosiváll,** MD, PhD, DSc Med, Medhabil (Professor) - Semmelweis University, Budapest, Hungary
- **Ernesto Sabath,** MD (Assistant Professor) - Hospital Ángeles Querétaro, Centro Sur Queretaro, Queretaro, Mexico
- **Sohail Abdul Salim,** MD (Adjunct Assistant Professor and Nephrologist) - University of Mississippi Medical Center, Jackson, Mississippi, USA and Southwest Kidney Institute, Phoenix, Arizona, USA (Clinical Sciences)
- **Satoshi Shimada,** MD, PhD (Instructor) - Medical College of Wisconsin, Milwaukee, Wisconsin, USA
- **Rui-Zhi Tan,** MD (Associate Professor) - Southwest Medical University, Luzhou, China
- **Kiyotaka Uchiyama,** MD, PhD (Associate Professor) - International University of Health and Welfare, Chiba, Japan
- **Michael E. Ullian,** MD (Professor) – Medical University of South Carolina, Charleston, South Carolina, USA (Clinical Sciences)
- **Xin Wang,** MD, PhD (Internal Medicine - Nephrology), Peking University People’s Hospital, Beijing, China
- **Wannasit Wathanavasin,** MD, MSc - Charoenkrung Pracharak Hospital, Bangkok, Thailand
- **Yonggui Wu,** MD (Professor) - Anhui Medical University, Hefei, China
- **Wan Xin,** MD (Nephrologist) – Nanjing Medical University, Nanjing, China
- **Zailing Xing,** MS, PhD(c) (Phd Candidate) - University of South Florida, Tampa, Florida, USA
- **Takahiro Yajima,** MD (Research Director) - Matsunami General Hospital, Gifu, Japan
- **Jianyun Yan,** PhD (Professor) - Zhujiang Hospital of Southern Medical University, Guangzhou, China
- **Selçuk Yüksel,** MD (Professor) - Çanakkale Onsekiz Mart University, Çanakkale, Türkiye
- **Mehtap Yuksel Egrilmez,** MD, PhD - Dokuz Eylul University, Izmir, Turkey
- **Hongliang Zhang,** MD, PhD (Director) - National Natural Science Foundation of China, Beijing, China

**Previous Editor:**

**William F. Finn,** MD - University of North Carolina at Chapel Hill, North Carolina, USA

